# Seropositivity of *Brucella* spp. and *Leptospira* spp. antibodies among abattoir workers and meat vendors in the city of Mwanza, Tanzania: A call for one health approach control strategies

**DOI:** 10.1371/journal.pntd.0006600

**Published:** 2018-06-25

**Authors:** Mariam M. Mirambo, Georgies F. Mgode, Zakaria O. Malima, Matata John, Elifuraha B. Mngumi, Ginethon G. Mhamphi, Stephen E. Mshana

**Affiliations:** 1 Department of Microbiology and Immunology, Weill Bugando School of Medicine, Catholic University of Health and Allied Sciences, Mwanza, Tanzania; 2 Pest Management Centre (SPMC), Sokoine University of Agriculture, Morogoro, Tanzania; 3 Department of Veterinary Pathology, Sokoine University of Agriculture, Morogoro, Tanzania; University of California Davis, UNITED STATES

## Abstract

**Introduction:**

Brucellosis and leptospirosis are among neglected tropical zoonotic diseases particularly in the resource limited countries. Despite being endemic in these countries, there is paucity of information on its magnitude. This study investigated seropositivity of *Brucella* spp. and *Leptospira* spp., and associated factors among abattoir workers and meat vendors in the city of Mwanza, Tanzania.

**Methodology:**

A community based cross-sectional study was conducted in Mwanza city from May to July 2017. Socio-demographic and other relevant information were collected. Detection of *Brucella* spp. and *Leptospira* spp. antibodies were done using slide agglutination test and microscopic agglutination test, respectively. Data were analyzed using STATA version 13 Software.

**Findings:**

A total of 250 participants (146 abattoir workers and 104 meat vendors) were enrolled with median age of 31 (IQR: 25–38) years. The overall, seropositivity of *Brucella* spp. antibodies was 48.4% (95% Cl: 42–54). Seropositivity of *B*. *abortus* was significantly higher than that of *B*. *melitensis* (46.0%, 95%Cl: 39–52 vs. 23.6%, 95% Cl: 18–28, P<0.001) while seropositivity of both species was 21.2% (95%Cl: 16–26). The seropositivity of *Leptospira* spp. was 10.0% (95% CI: 6–13) with predominance of *Leptospira kirschneri* serovar Sokoine which was detected in 7.2% of the participants. Being abattoir worker (OR: 2.19, 95% CI 1.06–4.54, p = 0.035) and long work duration (OR: 1.06, 95%CI: 1.01–1.11, p = 0.014) predicted presence of both *B*.*abortus* and *B*. *melitensis* antibodies. Only being married (p = 0.041) was significantly associated with seropositivity of *Leptospira* spp. Primary education was the only factor independently predicted presence of *Brucella* spp. antibodies among abattoir workers on sub-analysis of occupational exposure. None of factors were found to be associated with presence of *Brucella* spp. antibodies among meat vendors on sub-analysis.

**Conclusion:**

Seropositivity of *B*.*abortus* antibodies among abattoir workers and meat vendors is high and seem to be a function of being abattoir worker, having worked for long duration in the abattoir and having primary education. In addition, a significant proportion of abattoir workers and meat vendors in the city was seropositive for *Leptospira kirschneri* serovar Sokoine. There is a need to consider ‘one health approach’ in devising appropriate strategies to control these diseases in the developing countries.

## Author summary

Brucellosis and leptospirosis are among neglected diseases in many low-income countries affecting both animals and human populations. Despite being common, the information on their distribution are scarce. In a view of that, this study investigated the proportion of participants with positive antibody test specifically for the two diseases among slaughter house workers and meat sellers in Mwanza city. The study involved 250 participants aged between 25 and 38 years. Overall, 48.4% of participants were *Brucella* spp. seropositive. Proportion of participants who were positive for *B*. *abortus* specific antibodies was higher than that of *B*. *melitensis* while 21.2% of them found to have antibodies for both species studied. Furthermore, about 10% of the participants had *Leptospira* antibodies. Being abattoir worker, long work duration and having primary education were associated with the presence of *Brucella* antibodies while only being married was associated with the presence of *Leptospira* antibodies. The findings from this study emphasize the need for multisectoral approach in devising control strategies for these pathogens.

## Introduction

Brucellosis and Leptospirosis are among neglected tropical diseases which are endemic in resource limited countries including those in the sub-Saharan African region [1–3]. They are major public health concern due to their epidemiological patterns which involves animal-human interfaces resulting into economic losses and sub-clinical infections among human population. In human, these infections present with nonspecific symptoms, as a result they are misdiagnosed with other febrile illnesses such malaria, typhoid fever and rheumatic fever [4].

Leptospirosis is worldwide distributed particularly in tropical and some temperate regions. It is an occupational disease affecting individuals working close with animals. Leptospirosis outbreaks often occur after floods whereby the infected urine from animals such as rodents, dogs and cattle easily contaminate the water and environment hence spread the infection to humans [5,6]. The annual incidence of human Leptospirosis is estimated to be 1.03 million cases worldwide with 58,000 deaths being attributed to the disease [7]. In East African region the annual incidence is estimated to be 25.6 cases per 100,000 population [7].

Brucellosis is a contagious bacterial zoonotic disease of public health importance. Abattoir workers and others that work closely with animals or animal products have a high risk of contracting the disease[8,9]. The disease is endemic in the south and the Central America, Mediterranean, Africa, Indian subcontinent, Asia, Arab peninsula and Middle East. The annual incidence is estimated to range from 214.4 to 1603.4 cases per 100,000 population [10–12]. In Tanzania, the prevalence has been reported to range from 0.7 to 23.9% among the high risk groups [13–18].

Livestock brucellosis and leptospirosis [3,19–21] are endemic in the lake zone that supply animals destined for slaughter in Mwanza city. Abattoir workers and meat sellers may be at high risk if biosafety measures are not in place. Despite livestock brucellosis and leptospirosis being common in Tanzania especially in the Lake Victoria zone, there is paucity of data regarding the seropositivity of these pathogens among human population in the city of Mwanza, Tanzania. This study was designed to provide baseline information regarding the seropositivity of these pathogens, the information that may be useful for designing control interventions and provide insights for future research in this area.

## Methods

### Study design, study setting and study population

This was a community based cross-sectional study (S1 File) that was conducted between May and June 2017 in the city of Mwanza, Tanzania. The study was conducted among Igoma abattoir workers and meat vendors in the city. The abattoir has 250 workers, and about 200 cows and 50 goats are slaughtered per day. The city of Mwanza is the second largest in Tanzania with a total population of 706,453 according to 2012 National census [22]. The city possesses two districts namely; Ilemela and Nyamagana with a total population of 343,001 and 363,452, respectively. The city depends on Igoma abattoir to supply meat to more than 90 meat selling shops in the city.

### Sample size estimation, recruitment of the study participants and sample collection

The sample size was estimated by using Kish Leslie formula (1965), using the *Brucella* spp. seropositivity of 14.1% from a previous study by Mngumi et *al*., [17]. The Minimum sample size obtained was 186, however a total of 250 participants were enrolled. After obtaining a written informed consent, participants working in abattoir and meat retail shops were serially enrolled. Socio-demographic and other relevant information were collected using interview standard questions (S2 File). Data collected included: age, sex, occupation (abattoir worker, retail meat seller), residence, education and work duration in years.

About 4 to 5 mls of venous blood was collected in a plain vacutainer tubes (Becton, Dickinson and Company, USA) and transported to the Catholic University of Health and Allied sciences (CUHAS) multipurpose laboratory within 4 hours of collection. The sera were extracted by centrifugation at 2500 rpm for 10 minutes, decanted into cryovials in duplicates and stored at -40°C until processing. One set of the samples was transported to the Pest management centre at the Sokoine University of Agriculture (SUA) whereby the detection of *Leptospira* spp. antibodies was made. The detection of specific *Brucella* spp. antibodies was done at Catholic University of Health and Allied Sciences (CUHAS).

### Detection of *Brucella* spp. and *Leptospira* spp. antibodies

Detection of specific *Brucella* antibodies for *B*. *abortus* and *B*. *melitensis* was done using commercial rapid agglutination test according to the manufacturer’s instructions. The Eurocell A was used for *B*. *abortus* and Eurocell M for *B*. *melitensis* (Euromedi equip LTD.UK). In each run the positive and negative control were used. Results were interpreted as positive if the agglutination reaction was similar to that of positive control. The test has been found to have sensitivity and specificity of 95% and 100%, respectively [23].

Regarding the detection of *Leptospira* spp. antibodies, local Leptospira serovars previously isolated from animals (domestic animals and rodents) in Tanzania namely: *Leptospira kirschneri* serovar Sokoine, *L*. *interrogans* serovar Lora, *L*. *kirschneri* serovar Grippotyphosa, *L*. *borgpetersenii* serovar Kenya and *L*. *interrogans* serovar Hebdomadis were selected and used in microscopic agglutination test (MAT)[24,25]. The selected serovars were cultured into fresh *Leptospira* Ellinghausen and McCullough, modified by Johnson and Harris (EMJH) culture medium incubated at 30°C for 4 to 7 days before using as live antigen in (MAT). Culture purity and density was checked using dark-field microscope whereby a density of 300×10^8^ Leptospires/ml was considered adequate for MAT. Serum samples were serially diluted with phosphate buffered saline (pH 7) in a ratio of 1:10–1:80 in U–bottomed microtiter plate and 50 μl was used in MAT. Prepared live Leptospires antigen (50 μl) was added to the diluted serum to give final dilutions of 1:20–1:160 (100 μl total volume) of serum-antigen mixture in each microtiter well. The first row was used for negative control while positive control was added in the same row as the sample. The plates with serum–antigen mixture were incubated at 30°C for 2–4 hours before being examined for agglutination under dark field microscope.

A sample was considered positive for a specific serovar if more than 50% of the microorganisms in the microtiter well were agglutinated at the titer of ≥ 1: 80.

### Data analysis and management

Data collected was entered into a Microsoft excel sheet then analyzed using STATA version 13 software. The categorical variables were presented as proportions while continuous variables (age and work duration) were summarized as median with interquartile ranges. Cross-tabulation was done to determine factors with collinearity using Pearson Chi squared test. The median age and median work duration of *Brucella* spp. seropositive and seronegative participants were compared by Wilcoxon Mann-Whitney / ranksum tests. Logistic regression model was used to determine factors associated with the presence of specific *Brucella* spp. and *Leptospira* spp. antibodies. Factors with p value of less than 0.2 on univariate analysis were subjected to multivariable regression analysis. Odds ratio (OR) and 95% Confidence interval (Cl) were noted. A P value of < 0.05 was considered statistically significant.

### Ethical considerations

The protocol for conducting this study was approved by the joint CUHAS/BMC research ethics and review committee with ethical clearance number CREC/336/2017. The permission was further granted by the city council director, village leaders and abattoir manager. Written informed consent was obtained from each participant prior recruitment to the study. All participants in the current study aged 18 years and above

## Results

### Socio demographic characteristics of the study participants

All 250 participants were available for analysis. The median age of the study participants was 31 (inter quartile range (IQR): 25–38) years. One participant was female (0.4%) and the majority 212 (84.8%) of the participants were from urban areas. The majority of the participants 192/250 (76.80%) were married. Out of 250 participants, only 51 (20.40%) attended secondary education as shown in Table 1.

**Table 1:** Sociodemographic characteristics of the study participants conducted from May to July 2017.

Variable	Frequency/median	Percent (%)
**Age(years**)	31 (IQR 25–38)	50
**Sex**		
Female	1	0.40
Male	249	99.60
**Occupation**		
Abattoir workers	146	58.40
Meat vendors	104	41.60
**Residence**		
Rural	38	15.20
Urban	212	84.80
**Marital status**		
Married	192	76.80
Single	58	23.20
**Education level**		
Primary	199	79.6
Secondary	51	20.40
**Work duration (years**)	6(IQR 3–10)	50

The median work duration (years) of those with low education was 7 (IQR) 3–12 and that of those with high education was 3 (IQR) 2–5, p<0.001.

### Seropositivity of *Brucella* spp. antibodies among study participants

Overall seropositivity of *Brucella* spp. antibodies was found to be 48.4% (121/250, 95% Cl: 42–54). Seropositivity of *B*. *abortus* and *B*. *melitensis* was found to be 46.0% (115/250, Cl: 39–52) and 23.6% (59/250, 95% Cl: 18–28), respectively while seropositivity of co-infection of *B*. *abortus* and *B*. *melitensis* was 21.2% (53/250, 95% Cl: 16–26)

### Factors associated with of *B*. *abortus* seropositivity

**Fig 1:**
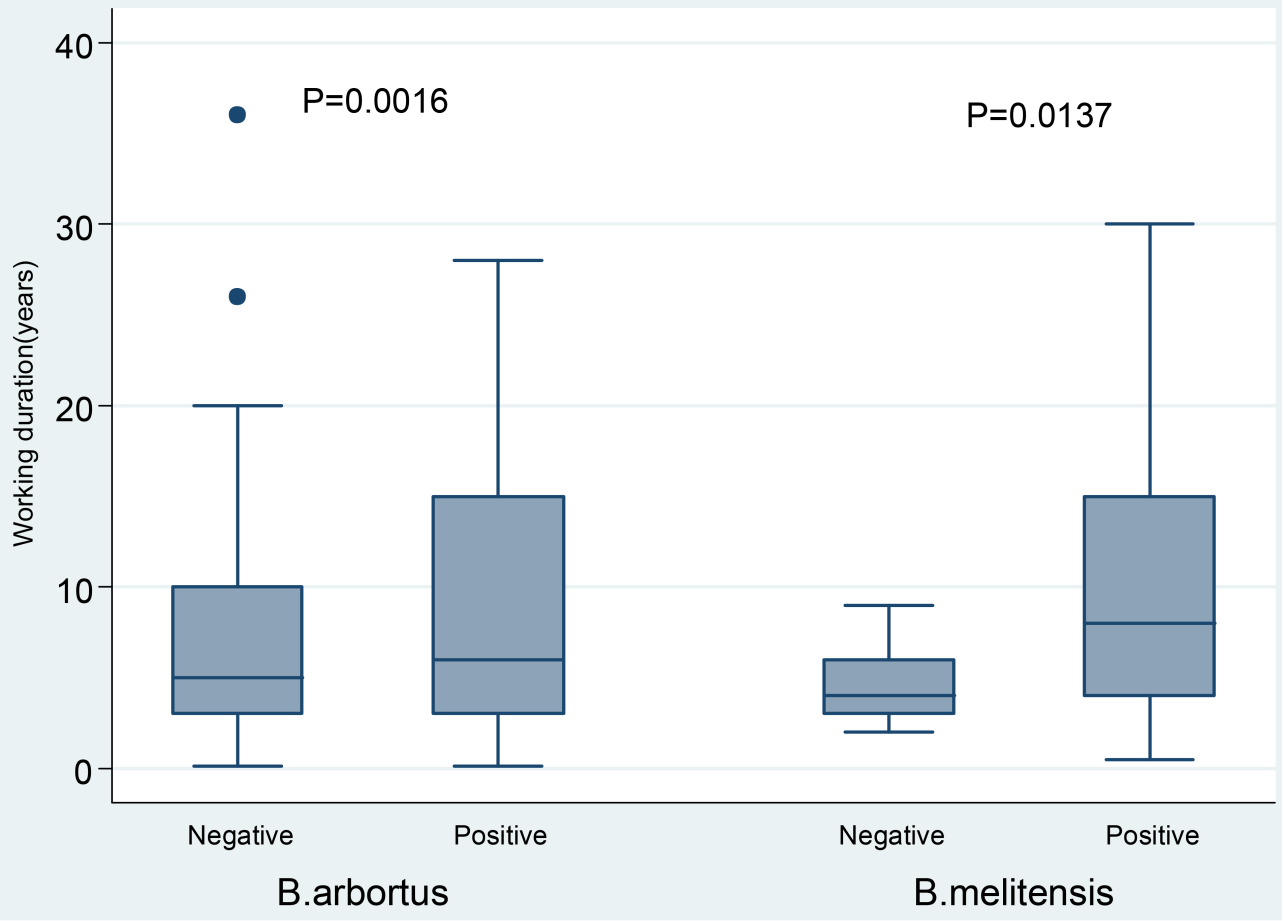
Boxplot showing the median work duration (years) between *Brucella* spp. seropositive and seronegative participants.

On univariate analysis, age (p = 0.029), residing in rural areas (p = 0.021), having primary education (p = 0.001), being abattoir worker (p<0.001) and work duration (p = 0.002) were significantly associated with the presence of specific *B*.*abortus* antibodies. The median work duration of *B*. *abortus* seropositive participants was 7(IQR4-15) years compared to 5(IQR 3–10) of those who were seronegative (p = 0.0016), Fig 1.

By multivariable logistic regression analysis, having primary education (OR:2.64, 95% CI:1.25–5.55, p = 0.011), being abattoir worker (OR:2.66,95% CI:1.49–4.77, p = 0.001) and having long work duration (OR:1.05, 95% CI:1.015–1.09, p = 0.041) were found to predict *B*. *abortus* seropositivity (Table 2).

**Table 2:** Factors associated with *B*.*abortus* seropositivity among study participants between May and July 2017.

Variables	*B*. *abortus* sero positivity (%)/Median	Univariate		Multivariable	
		Chi-square test/ Mann Whitney Ranksum test	P-Value	OR (95%Cl)	P-Value
**Age (years)**	33(IQR25-39)		0.029*		
**Marital status**					
Single(58)	19(32.8)				
Married(192)	96(50)	5.33	0.021	1.41(0.69–2.85)	0.346
**Residence**					
Urban(212)	91(42.9)				
Rural (38)	24(63.2)	5.31	0.021	1.53(0.69–3.35)	0.294
**Education level**					
Secondary(51)	13(25.5)				
Primary(199)	102(51.3)	10.85	0.001	2.63(1.25–5.55)	0.011
**Occupation**					
Meat vendors (104)	34(32.7)				
Abattoir workers (146)	81(55.5)	12.70	0.000	2.66(1.49–4.77)	0.001
**Accidents**					
NO(32)	13(40.6)				
YES(218)	102(46.8)	0.43	0.514		
**Fluid splash**					
NO(156)	71(45.5)				
YES(94)	44(46.8)	0.04	0.842		
**Bruises**					
NO(63)	27(42.9)				
YES(187)	88(47.1)	0.33	0.563		
**Work duration (years)**	5(IQR 3–10)		0.002*	1.05(1.00–1.09)	0.041

* Wilcoxon Mann-Whitney / ranksum tests, Age was not subjected on multivariable analysis due to its collinearity with work duration

### Factors associated with *B*. *melitensis* seropositivity

By univariate analysis, residing in rural areas (p = 0.037), being abattoir worker (p = 0.023) and long work duration (p = 0.014) were significantly associated with *B*. *melitensis* seropositivity. However, only long work duration (OR: 1.05, 95% CI: 1.00–1.10, p = 0.024) was found to predict *B*. *melitensis* seropositivity on multivariable logistic regression analysis (Table 3).

**Table 3:** Factors associated with *B*.*melitensis* seropositivity among study participants between May and July 2017.

Characteristics	*B*. *melitensis* seropositivity	Univariate		Multivariable	
		Chi-square test/ Mann Whitney Ranksum test	P-Value	OR (95%Cl)	P-Value
**Age(years)**	33(IQR24-40)		0.265*		
**Marital status**					
Single(58)	11(18.97)				
Married(192)	48(25)	0.90	0.343		
**Residence**					
Urban(212)	45(21.23)				
Rural(38)	14(36.84)	4.36	0.037	1.73(0.78–3.81)	0.174
**Education level**					
Secondary(51)	10(19.61)				
Primary(199)	49(24.62)	0.57	0.452		
**Occupation**					
Meat vendors (104)	17(16.35)				
Abattoir workers(146)	42(28.77)	5.20	0.023	1.84(0.93–3.63)	0.078
**Accidents**					
NO(32)	6(18.75)				
YES(218)	53(24.31)	0.48	0.489		
**Fluid splash**					
NO(156)	34(21.79)				
YES(94)	25(26.6)	0.75	0.387		
**Bruises**					
NO(63)	12(19.05)				
YES(187)	47(25.13)	0.97	0.325		
**Work duration(years**)	8(IQR 4–15)		0.014*	1.052(1.00–1.10)	**0.024**

* Wilcoxon Mann-Whitney / ranksum tests

### Factors associated with seropositivity of both *B*.*abortus* and *B*. *melitensis* specific antibodies

On univariate analysis residing in rural areas (p = 0.030), being abattoir worker (p = 0.012) and long work duration (p = 0.004) were significantly associated presence antibodies for both species. By multivariable logistic regression analysis; being abattoir worker (OR: 2.19, 95% CI: 1.06–4.54, p = 0.035) and having long work duration (OR: 1.06, 95% CI: 1.01–1.11, p = 0.014) were found to predict presence of antibodies for both species (*B*. *abortus* and *B*. *melitensis)* (Table 4).

**Table 4:** Factors associated with presence of antibodies for both species (*B*. *melitensis* and *B*. *abortus*) among study participants between May and July 2017.

Characteristic	Sero positivity of both species	Univariate		Multivariable	
		Chi-square test/ Mann Whitney Ranksum test	P-Value	OR (95%Cl)	P-Value
**Age**	34(IQR25-40)		0.148*		
Marital status					
Single(58)	8(13.79)				
Married (192)	45(23.44)	2.48	0.120		
**Residence**					
Urban (212)	40(18.87)				
Rural (38)	13(34.21)	4.54	0.030	1.67(0.74–3.78)	0.217
**Education level**					
Secondary (51)	7(13.73)				
Primary (199)	46(23.12)	2.14	0.140	1.51(0.61–3.76)	0.374
**Occupation**					
Meat vendors (104)	14(13.46)				
Abattoir workers(146)	39(26.71)	6.38	0.012	2.19(1.06–4.54)	0.035
**Accidents**					
NO(32)	6(18.75)				
YES(218)	47(21.56)	0.13	0.720		
**Fluid splash**					
NO(156)	30(19.23)				
YES(94)	23(24.47)	0.96	0.330		
**Bruises**					
NO(63)	10(15.87)				
YES(187)	43(22.99)	1.43	0.230		
**Work duration(years**)	8(IQR4-15)		0.004*	1.06(1.01–1.11)	0.014

* Wilcoxon Mann-Whitney / ranksum tests, Age was not subjected on multivariable regression analysis due to its collinearity with work duration

### Sub analysis of predictors of *Brucella* spp. seropositivity among the two occupation groups

When sub analysis was done among the two groups (abattoir workers and meat vendors) the following was observed. Among the variables tested in the abattoir workers group for *B*. *abortus*; increased in age (p = 0.001), long work duration (p = 0.004), being married (p = 0.007) and having primary education (p<0.001) were significantly associated with *B*. *abortus* seropositivity. By multivariable logistic regression analysis only having a primary education (OR: 3.77, 95% CI: 1.45–9.76, p = 0.006) was found to predict *B*. *abortus* seropositivity. Regarding *B*. *melitensis*. only long work duration (p = 0.013) was significantly associated with *B*. *melitensis* seropositivity. By multivariable logistic regression analysis none of the factors was found to predict *B*. *melitensis* seropositivity. Primary education (OR: 2.93, 95% CI: 1.19–7.23, p = 0.019) independently predicted *Brucella* spp. seropositivity. None of the factors tested was found to be associated with *Brucella* spp. seropositivity among meat vendors. (S3 File)

### Seropositivity of *Leptospira* spp. antibodies and associated factors among abattoir workers and meat vendors in Mwanza city

**Fig 2:**
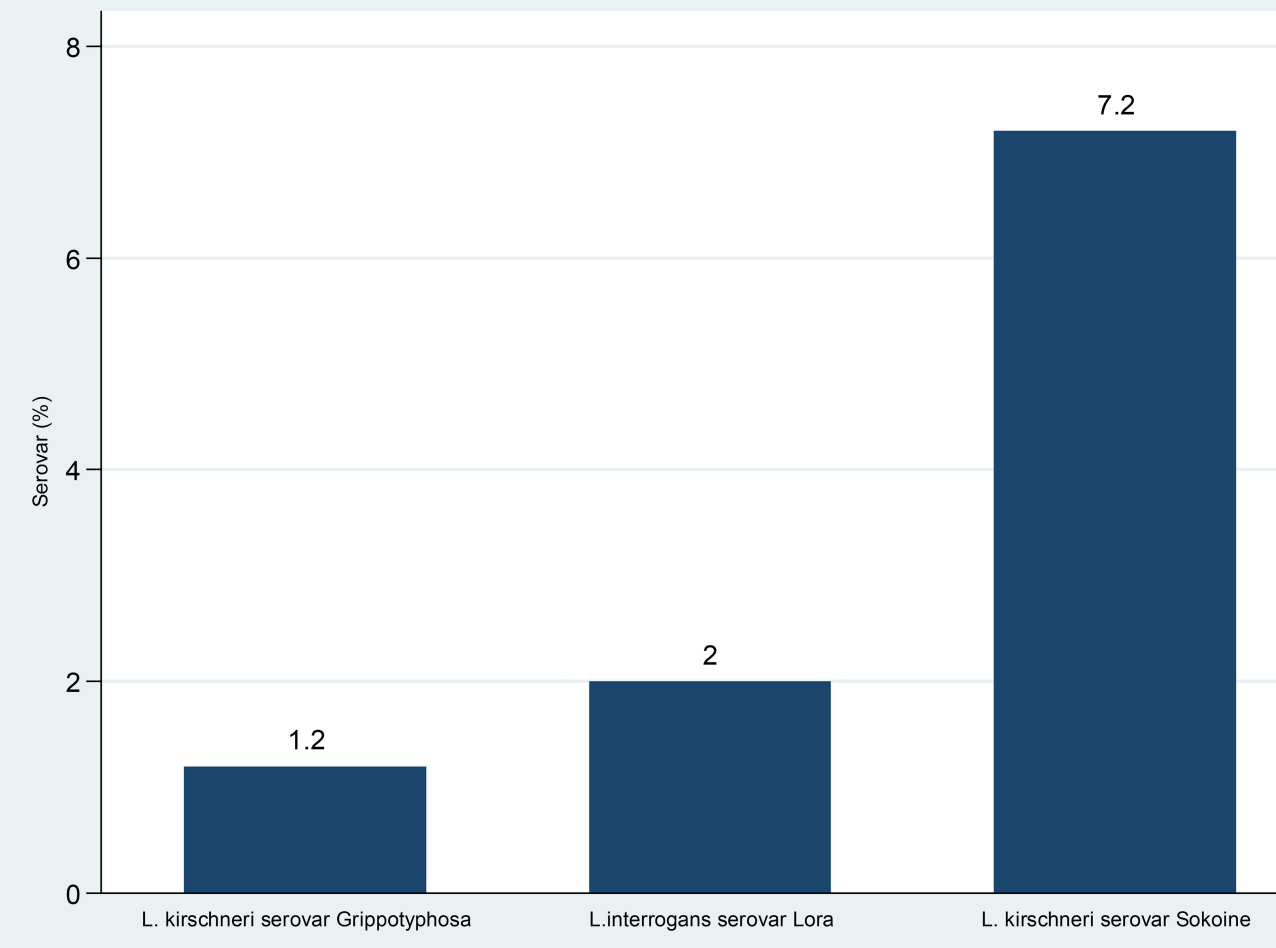
Seropositivity of *Leptospira* serovars among abattoir workers and meat vendors in Mwanza city.

Overall seropositivity of *Leptospira* spp. antibodies was found to be 26/250 (10.0%, 95% CI: 6–13). When categorized by occupation, there was no significant difference on seropositivity among abattoir workers and meat vendors (11/46(7.7%) vs. 14/104(13.5%), p = 0.124). Among the five *Leptospira* serovars tested, the most prevalent was *Leptospira kirschneri* serovar Sokoine (7.2%), followed by *L*. *interrogans* serovar Lora (2.0%) and *L*. *kirschneri* serovar Grippotyphosa (1.2%) (Fig 2). Other serovars tested were *L*. *borgpetersenii* serovar Kenya and *L*. *interrogans* serovar Hebdomadis in which none of the samples were found to be seropositive. Among the factors tested, only being married was significantly associated with seropositivity of *Leptospira* spp. (p = 0.041).

## Discussion

This is the first study to assess the seropositivity of the common *Brucella* spp., and Leptospira serovars circulating in Mwanza city among abattoir workers and meat vendors. The overall seropositivity of *Brucella* spp. was found to be high compared to the previous study conducted in North Karnataka, India among individuals with close contacts with animals[26]. In the contrary, the reported seropositivity in this study was indeed higher than what has been reported in other studies in Tanga city, Tanzania, Uganda, Ethiopia, Nigeria and Saudi Arabia which reported seropositivity of 5.5%, 10.0%, 4.7%, 24.1%, and 35.7%, respectively [16,27–30]. The difference could be explained by the difference in geographical locations, study population, specificity and sensitivity of the test used in different studies. In the current study Euro cell agglutination test was used with sensitivity and specificity of 95% and 100%, respectively [23].

In the current study, among the two *Brucella* spp. tested, the predominant species was *B*. *abortus*. This is inconsistent with previous study conducted in Sengerema district among agro-pastoral communities which reported high seropositivity of *B*. *melitensis* among agropastoralists who were not abattoir workers [17]. The possible explanation for the difference could be due to the occupational exposure to cattle which are the primary host of *B*. *abortus* and the most slaughtered animals in the city abattoir compared to sheep and goats which are more likely to be infected with *B*. *melitensis*. Our data indicated that individuals working at the city abattoir are more exposed to *B*. *abortus* infection than *B*. *melitensis* infection comparable to the previous study [16]. Regarding the magnitude of brucella antibodies for both species studied, in the current study, it was found to be significantly high compared to the previous study by Mngumi et al [17]. The possible explanation could be due differences in study population and duration of exposure of the risk factors.

In this study being abattoir worker was found to predict *Brucella* spp. seropositivity which is similar to the previous studies [30,31]. This could be explained by the fact that these individuals are more exposed to animals especially fetuses during slaughtering process hence they are at more risk to contract infection than meat vendors. Another factor which was found to predict *Brucella* spp. seropositivity was long work duration. This has been also observed in the previous study [31]. The possible explanation is the fact that those who worked in the abattoir for longer period are more likely to be exposed than those with short work duration. As in the previous report in Pakistan, using education levels as denominator, significantly high proportion of those with low education had *Brucella* spp. antibodies [31]. Having low education level might be associated with assignment to high risk tasks which might lead to frequent exposure to *Brucella* spp. Furthermore in the current study those with low education had significantly longer work duration in abattoir than those with high education level.

Regarding *Leptospira* spp. seropositivity, the overall seropositivity of *Leptospira* serovars in this study was almost similar to 9.5% which was reported among slaughter house workers in New Zealand[32]. In comparison to previous studies conducted in Katavi Tanzania among agropastoralists which reported the seropositivity of 29.9%, the observed seropositivity in the current study is significantly low[33]. In addition, compared to the study in Egypt[34] in which 16% of febrile patients had *Leptospira* antibodies, the observed seropositivity is also significantly low. The possible explanations to these variations could be due to different diagnostic techniques. The current study used MAT which has specificity and sensitivity of 95.7% and 55.3%, respectively [35] while other studies which reported high seropositivity used ELISA techniques which has been found to have sensitivity of >85%. Another possible explanation could be different in geographical and climatic conditions as well as study populations[36].

In the contrary, the seropositivity reported in this study is significantly higher than seropositivity of 1.1% and 0.66% reported in Reunion Island among general population[37,38]. The difference could be explained by the difference in the population studied, the current study investigated the seropositivity among high risk groups.

Among the serovars tested, the predominant serovar was *Leptospira kirschneri* serovar Sokoine, followed by *L*. *interrogans* serovar Lora and *L*. *kirschneri* serovar Grippotyphosa. This observation is similar to previous study conducted in Morogoro and Katavi among human population [33,39]. Further studies to explore more on the common serovars are recommended in Mwanza so as to get clear understanding of the common serovars circulating in the city. In the current study being married was significantly associated with leptospirosis. This observation is similar to the previous study conducted in Thailand [40]; no clear explanation could be established emphasizing the need for more studies to explore this factor. High number of married participants could also explain such observation in the current study.

### Limitation

Despite high specificity of serological test used in the current study, *B*. *abortus and B*. *melitensis* antigens are not specific when it comes to antigen-antibody assays because these two spp are more than 95% in structural homology. It should be noted that this study was done in the specific groups therefore results cannot be generalized to the general population of Mwanza city and being a cross-sectional study the trend of the outcome by time could not be established. In addition, the high seropositivity of *Brucella* spp. might be due to cross-reactivity of Brucella antigens with varieties *Enterobactericeae* antibodies. Brucella species identification through serology is markedly affected by cross reactions[41] and this is associated with high false positive.

### Conclusion and recommendations

*B*. *abortus* seropositivity among abattoir workers in Mwanza city is alarmingly high and is predicted having long work duration and having primary level of education. In addition, a significant proportion of this population is seropositive to *Leptospira kirschneri serovar* Sokoine. Being important zoonoses and neglected tropical diseases, there is a need to emphasize on biosafety measures during slaughtering, surveillance strategies, and treatment across the country particularly in high risk groups. Moreover, this calls for the need to adopt “One Health Approach” in controlling these diseases across the country. Further studies focusing on molecular detection of the pathogens to provide opportunities for understanding the infection patterns and the epidemiological implications of the pathogens to the high risk communities are highly warranted.

## Supporting information

S1 File Interview standard guide questions. (PDF)

S2 File Checklist: STROBE checklist. (PDF)

S3 File Tables of sub-analysis. (PDF)
