# Supplementary material for: Seropositivity of Brucella spp. and Leptospira spp. antibodies among abattoir workers and meat vendors in the city of Mwanza, Tanzania: A call for one health approach control strategies
Source: PLoS Negl Trop Dis. 2018 Jun 25;12(6):e0006600. doi: 10.1371/journal.pntd.0006600 (PMC6034905; doi:10.1371/journal.pntd.0006600)
Supplement: S1 File — (PDF) [file pntd.0006600.s001.pdf]

INTERVIEW GUIDE QUESTIONS

	**PARTICIPANT'S PARTICULARS**	
NO	**Question**	**Response**
1.	Date	____/____/20____
2.	Study number	__________________
3.	Mobile phone number	LB-________________
4.	Age	________________
5.	Sex	1. Male<br>2. Female
6.	Residence (mention place) __________________	1. Urban<br>2. Rural<br>3. Periurban
7.	Marital status	1. Married<br>2. Single<br>3. Widowed<br>4. Divorced
8.	Education level	1. No formal education<br>2. Primary<br>3. Secondary<br>4. Tertiary
9.	Occupation	1. Meat vendor<br>2. Abattoir worker
10.	If you re abattoir worker/meat seller, which task(s) are you involved in?	1. Unloading/moving animals<br>2. Cattle slaughterer<br>3. Goat/sheep slaughterer<br>4. Cleaner; floor, surfaces<br>5. Cleaning/washing equipments, gears etc.<br>6. Sewage system worker<br>7. Preparation of carcasses<br>8. Meat packer<br>9. Delivery driver<br>10. Handling byproducts (hides, skin, intestines etc.)<br>11. Weigh, wrap, and display cuts of meat<br>12. Other(specify)…………………….....<br>13. N/A
	**ASSOCIATED FACTORS**	
11.	For how long have you been working here?	Years__________or months______

12.	Have you been consuming raw meat/blood and/or unpasteurized milk?	1. YES<br>2. NO
13.	Do you normally wash fruit/vegetables before consumption?	1. YES<br>2. NO
14.	Do you have a habit of eating during work/breaks?	1. YES<br>2. NO
15.	Do you have habit of washing hands before and after eating at work?	1. YES<br>2. NO
16.	Do you always wear protective gears at work?	1. YES<br>2. NO
17.	If YES which one?	1. Coat<br>2. Gloves<br>3. Apron<br>4. Plastic boots<br>5. Cap<br>6. Face mask<br>7. Other(specify)…………………………
18.	How often do you clean your protective gears?	1. Everyday<br>2. Once a week<br>3. Twice a week<br>4. >2 a week
19.	House with sewage system (public service/septic tank)?	1. YES<br>2. NO
20.	Keeping pets (dogs/cats) at home?	1. YES<br>2. NO
21.	Are there any abandoned plots near your house?	1. YES<br>2. NO
22.	Do you have direct contact with blood/organs in the slaughterhouse?	1. YES<br>2. NO
23.	Do you have any contact with animal urine?	1. YES<br>2. NO
24.	Have you ever suffered from work related accident?	1. YES<br>2. NO
25.	If YES which one?	1. Fluid/blood splash<br>2. Cut/puncture wounds<br>3. Bruises<br>4. Other(specify)………………..
26.	Have you been excluded from work when getting wounds/bruisers?	1. YES<br>2. NO
27.	Have you been working in farms previously?	1. YES<br>2. NO
28.	Have you ever helped animal births?	1. YES<br>2. NO

29.	If YES, did you wear any protective gears?	1. YES<br>2. NO
30.	Do you keep clothes clean after work?	1. YES<br>2. NO
31.	Do you wash hands after animal contact?	1. YES<br>2. NO
32.	Are you working with bare feet?	1. YES<br>2. NO
33.	Have you ever been splashed with infected fluids (blood, urine, etc.)?	1. YES<br>2. NO
34.	Are there any rodents at home?	1. YES<br>2. NO
35.	Are you involved in paddy cultivation?	1. YES.<br>2. NO

****
