# Supplementary material for: Seropositivity of Brucella spp. and Leptospira spp. antibodies among abattoir workers and meat vendors in the city of Mwanza, Tanzania: A call for one health approach control strategies
Source: PLoS Negl Trop Dis. 2018 Jun 25;12(6):e0006600. doi: 10.1371/journal.pntd.0006600 (PMC6034905; doi:10.1371/journal.pntd.0006600)
Supplement: S2 File — (PDF) [file pntd.0006600.s002.pdf]

Predominance of Brucella abortus and Leptospirosis among abattoir workers and meat vendors in the city of Mwanza, Tanzania: A call for one health approach control strategies

STROBE Statement—Checklist of items that should be included in reports of ***cross-sectional studies***

	Item No	Recommendation	Location
**Title and abstract**	1	(*a*) Indicate the study's design with a commonly used term in the title or the abstract	The design is mentioned in the abstract methodology section
		(*b*) Provide in the abstract an informative and balanced summary of what was done and what was found	Abstract
**Introduction**			
Background/rationale	2	Explain the scientific background and rationale for the investigation being reported	Background section
Objectives	3	State specific objectives, including any pre-specified hypotheses	Background: Paragraph 4
**Methods**			
Study design	4	Present key elements of study design early in the paper	Methods: Study design, study setting and study population
Setting	5	Describe the setting, locations, and relevant dates, including periods of recruitment, exposure, follow-up, and data collection	Methods: Study design, study setting and study population
Participants	6	(*a*) Give the eligibility criteria, and the sources and methods of selection of participants	See: Sample size estimation, recruitment of the study participants and sample collection
Variables	7	Clearly define all outcomes, exposures, predictors, potential confounders, and effect modifiers. Give diagnostic criteria, if applicable	Data analysis section
Data sources/ measurement	8*	For each variable of interest, give sources of data and details of methods of assessment (measurement). Describe comparability of assessment methods if there is more than one group	Detection of brucella and Leptospiral antibodies section
Bias	9	Describe any efforts to address potential sources of bias	Sample size estimation, recruitment of the study participants and sample collection:

			Paragraph 1 and Paragraph 1 Data analysis section
Study size	10	Explain how the study size was arrived at	Sample size estimation, recruitment of the study participants and sample collection section
Quantitative variables	11	Explain how quantitative variables were handled in the analyses. If applicable, describe which groupings were chosen and why	Data analysis section
Statistical methods	12	(*a*) Describe all statistical methods, including those used to control for confounding	Data analysis
		(*b*) Describe any methods used to examine subgroups and interactions	Data analysis
		(*c*) Explain how missing data were addressed	-
		(*d*) If applicable, describe analytical methods taking account of sampling strategy	
		(*e*) Describe any sensitivity analyses	Detection of brucella and Leptospiral antibodies: Paragraph 1
**Results**			
Participants	13*	(a) Report numbers of individuals at each stage of study—eg numbers potentially eligible, examined for eligibility, confirmed eligible, included in the study, completing follow-up, and analysed	Socio demographic characteristics of the study participants
		(b) Give reasons for non-participation at each stage	
		(c) Consider use of a flow diagram	-
Descriptive data	14*	(a) Give characteristics of study participants (eg demographic, clinical, social) and information on exposures and potential confounders	Socio demographic characteristics of the study participants
		(b) Indicate number of participants with missing data for each variable of interest	-
Outcome data	15*	Report numbers of outcome events or summary measures	Prevalence of Brucella spp. antibodies among study participants and Seroprevalence of Leptospira and associated factors among abattoir workers and meat vendors in Mwanza city

Main results	16	(*a*) Give unadjusted estimates and, if applicable, confounder-adjusted estimates and their precision (eg, 95% confidence interval). Make clear which confounders were adjusted for and why they were included	Data analysis section<br>Table 2, table 3 and table 4
		(*b*) Report category boundaries when continuous variables were categorized	Tables 1, table 2, table 3
		(*c*) If relevant, consider translating estimates of relative risk into absolute risk for a meaningful time period	
Other analyses	17	Report other analyses done—eg analyses of subgroups and interactions, and sensitivity analyses	
**Discussion**			
Key results	18	Summarise key results with reference to study objectives	Discussion: Paragraph 1 and 2
Limitations	19	Discuss limitations of the study, taking into account sources of potential bias or imprecision. Discuss both direction and magnitude of any potential bias	Limitation section
Interpretation	20	Give a cautious overall interpretation of results considering objectives, limitations, multiplicity of analyses, results from similar studies, and other relevant evidence	Discussion paragraph 1, 2,3
Generalisability	21	Discuss the generalisability (external validity) of the study results	Limitation section
**Other information**			
Funding	22	Give the source of funding and the role of the funders for the present study and, if applicable, for the original study on which the present article is based	Acknowledgement section second line

*Give information separately for exposed and unexposed groups.

Note: An Explanation and Elaboration article discusses each checklist item and gives methodological background and published examples of transparent reporting. The STROBE checklist is best used in conjunction with this article (freely available on the Web sites of PLoS Medicine at http://www.plosmedicine.org/, Annals of Internal Medicine at http://www.annals.org/, and Epidemiology at http://www.epidem.com/). Information on the STROBE Initiative is available at www.strobe-statement.org.
