# Supplementary material for: Seropositivity of Brucella spp. and Leptospira spp. antibodies among abattoir workers and meat vendors in the city of Mwanza, Tanzania: A call for one health approach control strategies
Source: PLoS Negl Trop Dis. 2018 Jun 25;12(6):e0006600. doi: 10.1371/journal.pntd.0006600 (PMC6034905; doi:10.1371/journal.pntd.0006600)
Supplement: S3 File — (PDF) [file pntd.0006600.s003.pdf]

S3 File. Tables of sub-analysis

Factors associated with *B. abortus* seropositivity among abattoir workers in Mwanza city between May and July 2017

**Characteristic**	***B. abortus* sero positivity**	**Univariate**		**Multivariable**	
		**Chi-square test/ Mann Whitney Ranksum test**	**P-Value**	**OR (95%Cl)**	**P-Value**
**Age**	32(IQR25-38)		0.001	1.04(0.98-1.10)	0.150
Marital status					
Single (36)	13(36.11)				
Married (110)	68(61.82)	1.00	0.007	1.57(0.61-4.01)	0.342
**Residence**					
Urban 109)	57(52.29)				
Rural (37)	24(64.86)	1.77	0.184	1.84(0.75-4.45)	0.177
**Education level**					
Secondary (33)	8(24.24)				
Primary (113)	73(64.60)	16.84	>0.001	3.77(1.45-9.76)	**0.006**
**Accidents**					
NO(20)	8(40.00)				
YES(126)	73(57.94)	2.25	0.134	1.17(0.39-3.48)	0.774
**Fluid splash**					
NO(67)	35(52.24)				
YES(79)	46(58.23)	0.52	0.468		
**Bruises**					
NO(42)	20(47.62)				
YES(104)	61(58.65)	1.47	0.225		
***Work duration(years)**	8(IQR:4-15)		0.003		

*Work duration has collinearity with age therefore it was not subjected in multivariate analysis

Factors associated with *Brucella* spp. seropositivity among abattoir workers in Mwanza city between May and July 2017

**Characteristic**	***Brucella* spp. sero positivity**	**Univariate**		**Multivariable**	
		**Chi-square test/ Mann Whitney Ranksum test**	**P-Value**	**OR (95%Cl)**	**P-Value**
**Age**	31(IQR:25-38)		0.005		
Marital status					
Single (36)	15(41.67)				
Married (110)	69(62.73)	4.92	0.026	1.38(0.57-3.31)	0.467
**Residence**					
Urban (109)	59(54.13)				
Rural (37)	25(67.57)	2.04	0.153	1.72(0.72-4.09)	0.217
**Education level**					
Secondary (33)	10(30.30)				
Primary (113)	74(65.49)	12.94	>0.001	2.93(1.19-7.23)	**0.019**
**Accidents**					
NO(20)	8(40.00)				
YES(126)	76(60.32)	2.91	0.088	1.36(0.45-4.05)	0.580
**Fluid splash**					
NO(67)	37(55.22)				
YES(79)	47(59.49)	0.27	0.603		
**Bruises**					
NO(42)	21(50.00)				
YES(104)	63(60.58)	1.36	0.242	1.56(0.70-3.45)	0.267
**Work duration(years)**	7.5(IQR:4-13.5)		0.011	1.05(0.97-1.13)	0.179

Factors associated with *Brucella* spp. seropositivity among meat vendors in Mwanza city between May and July 2017

**Characteristic**	***Brucella* spp. sero positivity**	**Univariate**	
		**Chi-square test/ Mann Whitney Ranksum test**	**P-Value**
**Age**	38(IQR:24-45)		0.235
Marital status			
Single (22)	7(31.82)		
Married (82)	30(36.59)	0.17	0.678
**Residence**			
Urban (103)	37(35.92)		
Rural (1)	0(0.00)	2.56	0.455
**Education level**			
Secondary (18)	6(33.33)		
Primary (86)	31(36.05)	0.04	0.827
**Accidents**			
NO(12)	5(41.67)		
YES(92)	32(34.78)	0.21	0.639
**Fluid splash**			
NO(27)	9(33.33)		
YES(77)	28(36.36)	0.08	0.777
**Bruises**			
NO(42)	21(50.00)		
YES(83)	29(34.94)	0.07	0.787
**Work duration(years**)	6(IQR:3-16)		0.205
